# Biomimetic Actuation and Artificial Muscle

**DOI:** 10.1155/2018/4617460

**Published:** 2018-06-14

**Authors:** Qingsong He, David Vokoun, Qi Shen

**Affiliations:** ^1^Nanjing University of Aeronautics and Astronautics, Nanjing, China; ^2^Academy of Sciences of the Czech Republic, Prague, Czech Republic; ^3^University of Nevada, Las Vegas, Las Vegas, NV, USA

In general, actuation technology reflects the progress in the development of mechanical systems. Originally, actuators were linked to steam engines, then several decades later to internal combustion engines and to electromotors, making mechanical systems increasingly more flexible, with higher energy utilization and convenience. However, the traditional actuation technologies (e.g., miniature motors and gear transmissions) cannot compete with the high flexibility, high redundancy, and large load-to-weight ratio found in animal muscular systems, which cause the performance of robots to fall behind that of the animals. Distributed actuation found in animal muscular systems is one of the main reasons of flexibility, high efficiency, and large energy density. Thus, the biomimetic actuators or artificial muscles with large displacements have drawn great attention in research and industrial application development.

For these reasons, a large number of scientific works are conducted and this special issue selects the related papers from a great number of high-quality candidates. In this special issue, some important aspects of biomechanics, biomimetic actuation, and artificial muscle and sensor are covered. This special issue starts with an investigation of the internal environment and structure of a *Gekko gecko*'s brain, going to a biomimetic beetle-inspired flapping air vehicle using ionic polymer-metal composite artificial muscle. Then in the third paper, we move to active tube-shaped actuator. With the fourth paper, we start to study computing method to determine the performance of the soft actuator. The fifth paper addresses the gait design and trajectory planning to further develop dynamic gait and reduce energy consumption of climbing robot. And a new high-reliable and lightweight embedded optical torque sensor is proposed for biomimetic robot arm in the sixth paper. Finally, a volumetric wear assessment methodology for polyethylene tibial knee inserts in total knee replacements is presented in the seventh paper. More in detail, a brief summary of each paper is as follows.

The first paper “Passive Cushiony Biomechanics of Head Protection in Falling Geckos” by H. Wang et al. used the magnetic resonance imaging technique to scan a *Gekko gecko*'s brain to study its internal environment and structure in order to understand the mechanism responsible for the self-protection in case of head impact. The results showed that the brain parenchyma was fully surrounded by the cerebrospinal fluid (CSF) in the skull. A succulent characteristic was presented, which meant the intracalvarium was significantly occupied by the CSF, up to 45% in volume. Then a simplified 3D finite element model was built, and a dynamic simulation was conducted to evaluate the mechanical property of this succulent characteristic during head impacts. These implied the succulent characteristics may play certain roles on the self-protection in case of head impact, which is adaptable to the *Gekko gecko*'s locomotion and behavior.

The paper “Biomimetic Beetle-Inspired Flapping Air Vehicle Actuated by Ionic Polymer-Metal Composite Actuator” by Y. Zhao et al. proposed a biomimetic flapping air vehicle by combining the superiority of ionic polymer-metal composite (IPMC) with the bionic beetle flapping principle. The blocking force was compared between casted IPMC and IPMC. The flapping state of the wing was investigated, and the maximum displacement and flapping angle were measured. The flapping displacement under different voltages and frequencies was tested. The flapping displacement of the wing and the support reaction force were measured under different frequencies by experiments. The experimental results indicate that the high voltage and low frequency would get large flapping displacement.

The third paper of this special issue “Active Tube-Shaped Actuator with Embedded Square Rod-Shaped Ionic Polymer-Metal Composites for Robotic-Assisted Manipulation” by Y. Wang et al. creates an active tube by integrating bar-shaped IPMC actuators into a soft silicon rubber structure. The authors modified the Nafion solution casting method and developed a complete sequence of fabrication process for bar-shaped IPMCs with square cross sections and four insulated electrodes on the surface. The silicon gel was cured at a suitable temperature to form a flexible tube using molds fabricated by 3D printing technology. By applying different voltages to the four electrodes of each IPMC bar-shaped actuator, complex multidegree of freedom bending motions of the active tube can be generated.

In the fourth paper “A Computing Method to Determine the Performance of an Ionic Liquid Gel Soft Actuator” by B. He et al., the authors model ionic liquid gel using Mooney-Rivlin model, capable of describing the nonlinear character of the mechanical response. Their model is validated by experimental stress-strain data obtained for a manufactured ionic liquid gel sample.

X. Li et al., in their paper “The Gait Design and Trajectory Planning of a Gecko-Inspired Climbing Robot,” analyzed the gait design and trajectory planning, in order to further develop dynamic gait and reduce energy consumption of climbing robot based on a model with a pendular waist and four active linear legs.

Y. Liu et al., in their paper “A Highly Reliable Embedded Optical Torque Sensor Based on Flexure Spring,” proposed a new high-reliable and lightweight embedded optical torque sensor for biomimetic robot arm enabling the torque measurement in joints, which can measure torque of the joint by detecting torsion of its elastic element.

Finally, in the paper “CMM-Based Volumetric Assessment Methodology for Polyethylene Tibial Knee Inserts in Total Knee Replacement,” W. Jiang et al. presented a new coordinate measuring machine- (CMM-) based methodology to determine volumetric material loss based on curve surface fitting without prewear data, CAD model, or original design of drawings. The results indicate that the methodology is adequate for clinically retrieved tibial inserts where no prewear data are provided. This technique can also be used for biotribological study of other polyethylene components, since wear and damage can be assessed visually and volumetrically.

This special issue aims to promote research and provide a platform for communication of novel ideas, theories, and technologies in biomimetic actuation, sensing, and materials. We hope that this special issue, the regular issue, and other special issues of this journal are beneficial and interesting for you.

*Qingsong He* *David Vokoun* *Qi Shen*

